# Smoking-Related Knowledge, Attitude, Social Pressure, and Environmental Constraints among New Undergraduates in Chongqing, China

**DOI:** 10.3390/ijerph120100895

**Published:** 2015-01-19

**Authors:** Xianglong Xu, Doris Yin Ping Leung, Bing Li, Pengfei Wang, Yong Zhao

**Affiliations:** 1 School of Public Health and Management, Chongqing Medical University, No.1 Yixueyuan Road, Yuzhong District, Chongqing 400016, China; E-Mails: xianglong1989@126.com (X.X.); wangpengfeiyong@126.com (P.F.); 2 The Nethersole School of Nursing, The Chinese University of Hong Kong, Hong Kong; E-Mail: dorisleung@cuhk.edu.hk; 3 School of the Second Clinical, Chongqing Medical University, No.1 Yixueyuan Road, Yuzhong District, Chongqing 400016, China; E-Mail: chongyilb@sina.cn

**Keywords:** undergraduates, smoking, attitude, environmental constraints, knowledge, social pressure

## Abstract

*Background*: Smoking has resulted in numerous deaths in China. Data indicate that 21% of college students in China are smokers. *Objective*: This study aimed to examine the smoking-related behaviors of undergraduates, as influenced by knowledge, attitude, social pressure, and environmental constraints. *Method*: A convenience sampling of 412 fresh undergraduates from two universities in the University Town in Chongqing, China was recruited. Chi-square tests were used to compare the smoking-related variables between smokers and non-smokers. Moreover, logistic regression was used to examine the factors that associated with smoking status in undergraduates. *Results*: Smokers and non-smokers differ in terms of knowledge, attitudes toward smoking, participation in tobacco promotional activities, and sources of social pressure. Logistic regression model identified that sex, living cost, five smoking-related attitudes of “Smoking is pleasurable, Smoking relaxes me, Smoking makes me look strong, Smoking is a waste of money, Smoking can help me study better”, the social pressure “Smoking brings comfort during celebration”, and the environmental constraints “How did you get your cigarettes in the past 30 days?” are significantly associated with smoking. *Conclusions*: The findings provide a better understanding of the epidemic of smoking among fresh undergraduates in Chongqing, China. This study provides more detailed consideration of the implications for the WHO Framework Convention on Tobacco Control (FCTC) policies, especially on restriction of retail sales outlets and tobacco promotion activities near universities in China.

## 1. Introduction

China is the largest producer and consumer of tobacco in the world, accounting for 40% of global cigarette production and consumption [1]. More than 300 million people smoke in China, and one million people die from tobacco-related diseases each year [2]. China has about 560 million adult non-smokers exposed to secondhand smoke, plus 180 million teenagers, a total of 740 million non-smokers are from exposure second-hand smoke [3]. Smoking prevention and control policies in several countries have focused on the youth because most smokers are believed to start smoking during adolescence. In China, a population-based survey showed that the smoking prevalence among individuals aged 15–24 was 17.9% [4]. Data indicate that 21% of college students in China are smokers, and that smoking is more prevalent in southwestern cities than in coastal cities [5]. Previous longitudinal studies have suggested that the process of becoming a regular smoker is not straightforward among college students [6]. As teens develop self-consciousness, their sense of independence and eagerness to break free from the expectations of adults is gradually enhanced. Thus, adolescents are vulnerable to negative peer influence, which can induce all types of health-risk behaviors [7]. New undergraduates are transitioning from puberty to young adulthood. This process could be disrupted if they fail to regulate their mental and physical health, particularly for young adults who left their homes and have to adapt to their new lives at the university. Previous study suggested that the time spent in college is conducive for developing the smoking habit [8]. However, previous studies lack evidence on the onset of smoking habits among Chinese and non-Western populations.

Adolescent smoking is associated with various factors, including demographic characteristics, interpersonal issues (e.g., social norms and social influence of smoking), and physical environmental factors (e.g., availability of cigarettes and space for smoking). The proximal and distal psychological risk factors of smoking behavior and intention vary based on the cultural context [9,10]. Proximal factors include peer influence, which is often expressed as peer pressure [11], and positive attitude toward smokers and smoking cohabitants [10,12]. Distal factors include emotional distress from having a reciprocal relationship with smoking behavior [13], such as social motives, effect and stress, knowledge about smoking, perceived benefits of smoking, risk perception of smoking, media, and tobacco advertising/promotion, and family and school environment [14,15,16]. Barriers or physical constraints are similarly related to the change in smoking behaviors among adolescents [17]. This study aims to examine the smoking-related behaviors of undergraduates, as influenced by knowledge, attitude, social pressure, and environmental constraints.

## 2. Methods

### 2.1. Study Design

A cross-sectional study was conducted on fresh undergraduates at Chongqing University Town, China. Chongqing University Town is located in the Shapingba district, about 15 km far from the downtown area of Chongqing. In 2010, there were more than 150,000 students in the 17 universities and colleges located in the Chongqing University Town. Each university/college in the University Town has comparable numbers of students. We chose students attending eight classes at the Grade One level from two universities, namely, Chongqing Normal University and Chongqing Medical University, by convenience sampling. Students from the two universities were chosen because they majored in education and medicine and hence are expected to be an important workforce in health education and promotion after their graduation.

### 2.2. Setting and Samples

Participants were all the 436 students attending the eight selected classes. A pilot study was conducted in November 2010 to test the feasibility of the proposed study. The questionnaire was administered to two groups of 40 undergraduate students; the first group comprised students from Chongqing Normal University and the other group from Chongqing Medical University. Based on the experience of the pilot study, students were contacted in their classroom before or after lectures by a trained student helper in the current study. The student helper explained the research objectives, and distributed the questionnaire to students who gave written consent to participate. The consented students were asked to complete the questionnaire in the classroom. Respondents required 12 to 15 min to complete the questionnaire. A total of 412 students responded which represents a response rate of 94.5%. Out of the collected 412 questionnaires, 23 responses were deleted because of missing data, thus resulting in a final sample of 389 (*i.e.*, response rate of 94.4%) for analysis.

### 2.3. Ethical Considerations

This study was approved by The University of Hong Kong. Written informed consent for processing personal data was obtained from all of the participants.

### 2.4. Instrument

The questionnaire was derived from previous studies on the National Youth Tobacco Survey in US and territorial-wide school-based survey in Hong Kong, and both aimed to understand youth tobacco use over a series of years [9,14,18,19]. The questionnaire was customized for the target population based on the pilot study and the final draft of the questionnaire was agreed after several discussions with expert and the pilot investigation. The collected information includes smoking status, demographic data and variables on smoking-related knowledge, participation in tobacco promotional activities, smoking-related attitude, social pressure and environmental constraints. Smoking status was measured by one item asking the respondents to indicate whether they had smoked before. Smokers were defined as those who had attempted to smoke even one or two cigarettes. Demographic data includes age, gender, living expenses (<¥600/¥600–800/>¥800**)**, and self-reported physical conditions (well/general/poor). Smoking-related knowledge was measured with six items on knowledge on smoking on health in general (three items) and knowledge of smoking contributing to an increased risk of common diseases (three items) [14]. Participation in tobacco promotion activities was measured by 11 items which asked the respondents to indicate whether they had participated in four main types of tobacco promotion in the past (four items) and the sources of tobacco advertising in the mass media (seven items). Smoking-related attitude was measured by their agreement on the statements (1) smoking is fun; (2) smoking is a type of self-presentation; (3) smoking relaxes nerves; (4) smoking makes you look tough; (5) feel mature; (6) gives you confidence; (7) smoking is a waste of money; (8) smoking can reduce weight; (9) perform better in sports; and (10) perform better in study on a 5-point Likert scale (“strongly agree” to “strongly disagree”) [9]. Social pressure was measured by items such as “Have you ever felt pressure to smoke from your friend?” on a 5-point Likert scale: “very often”, “often”, “sometimes”, “a few times”, and “never”, and questions like “Does smoking cigarettes help people feel more or less comfortable at celebrations, parties, or in other social gatherings?” [10]. Environmental constraints include the availability of cigarettes and space, each of these two aspects was measured by two items [20,21]. We asked all the students to complete the items on environmental constraints because students in the sample lived on campus and thus it is possible that non-smokers also buy cigarettes for their peers on request.

### 2.5. Data Analysis

The data was carefully reviewed prior to their entry into the database using EPI Data 3.1 software. Data analysis was performed using statistical analysis system software (version 9.1; SAS Institute, Cary, NC, USA) after meticulous data sorting and cleaning. The characteristics of the participants were summarized using either means and standard deviations or frequencies and percentages, and were presented using descriptive analysis (means, standard deviations, and percentages). Chi-square tests were employed for comparisons when appropriate. Logistic regression was performed to examine the factors that affect undergraduate smoking. We included variables that have a *p*-value < 0.2 in the bivariate analysis with “smoking status” as the dependent variable in the regression model with backward elimination to retain those factors that were still significant. The independent variable “How did you obtain cigarettes in the past 30 days?” was recorded into 2 categories: 1 = “Buy cigarettes” and 0 = “Do not buy any cigarettes” due to zero cell size in one of the responses for selection. The threshold for statistical significance was established at the 0.05 level in the logistic regression.

## 3. Results

### 3.1. Characteristics of Participants

The participants comprised 196 males and 193 females. Their ages ranged from 16 to 23 years. The sample consisted of 85 (21.85%) smokers and 304 (78.15%) non-smokers, and the average age of smokers was 20.7 ± 1.3 years, whereas that of non-smokers was 19.4 ± 1.3 years. The differences between the age of smokers and non-smokers were statistically significant (*p* < 0.0001). The comparison between the physical condition of smokers and non-smokers yielded no statistically significant difference (*p* = 0.1799) (see Table 1).

**Table 1:** Demographic characteristics of participants (*N* = 389).

Variable	Smokers		Non-Smokers		Statistical Test
	*N* (%)	Mean ± SD	*N* (%)	Mean ± SD	
Sex					*χ^2^* = 74.4977, *p* < 0.0001
Male	78 (91.8)	--	118 (38.8)	--	
Female	7 (8.2)	--	186 (61.2)	--	
Age (16–23)	--	20.7 ± 1.3	--	19.4 ± 1.3	*t* value = 8.23, *p* < 0.0001
Physical conditions					*χ^2^* = 3.4312, *p* = 0.1799
Well	24 (28.2)	--	60 (19.7)	--	
General	52 (61.2)	--	217 (71.4)	--	
Poor	9 (10.6)	--	27 (8.9)	--	
Living cost					*χ^2^* = 5.9387, *p* = 0.0513
<**¥**600	23 (27.1)	--	126 (41.4)	--	
**¥**600–**¥**800	33 (38.8)	--	99 (32.6)	--	
>**¥**800	29 (34.1)	--	79 (26.0)	--	

### 3.2. Smoking-Related Knowledge

Table 2 shows the differences in knowledge about smoking between smokers and non-smokers. The perception of the dangers of cigarette use (“smoking is harmful,” “smoking is harmful to children and infants,” “smoking could cause lung cancer,” and “passive smoking could cause lung cancer”) was significantly higher for non-smokers than smokers (*p* < 0.05). However, no significant difference was observed between smokers and non-smokers with regard to the perception of “smoking resulting in weight gain or loss” (*p* = 0.9614) and “smoking causing heart disease or chronic heart diseases” (*p* = 0.9054).

**Table 2:** Comparison of Smoking-related knowledge between smokers and non-smokers (*N*, %).

Variable	Smokers	Non-Smokers	Statistical Test
Smoking is harmful.	67 (78.8)	282 (92.8)	*χ^2^* = 13.9913, *p* = 0.0002
Smoking can help one gain or lose weight.	6 (7.1)	21 (6.9)	*χ^2^* = 0.0023, *p* = 0.9614
Smoking can cause heart diseases or CHD.	50 (58.8)	81 (59.5)	*χ^2^* = 0.0141, *p* = 0.9054
Passive smoking is harmful to children and infants.	69 (81.2)	280 (92.1)	*χ^2^* = 8.6001, *p* = 0.0034
Smoking could cause lung cancer.	64 (75.3)	261 (85.9)	*χ^2^* = 5.3901, *p* = 0.0203
Passive smoking could cause lung cancer.	64 (75.3)	260 (85.5)	*χ^2^* = 4.9971, *p* = 0.0254

### 3.3. Tobacco Promotion and Advertisement

Table 3 lists the differences between the participation of smokers and non-smokers in tobacco promotion activities. Compared to non-smokers, smokers were more likely to admit to have exchanged a cigarette case for an entertainment ticket (14.1% *vs.* 4.6%, *p* = 0.0019), to express affirmation in collecting a gift or on-sale goods using a cigarette case non-smokers (3.0%) (9.4% *vs.* 3.0%, *p* = 0.0164), and to have joined an activity hosted by a cigarette company than non-smokers (3.6%) (9.4% *vs.* 3.6%, *p* = 0.0425). Moreover, more smokers (15.3%) accepted free cigarettes during promotional activities compared with non-smokers (5.6%) (*p* = 0.0030). The number of non-smokers exposed to tobacco advertisements through billboards (*p* = 0.0323), newspapers (*p* = 0.0497), and television (*p* = 0.0074) was significantly larger compared with smokers.

**Table 3:** Comparison of participation in tobacco promotional activities between smokers and non-smokers (*N*, %).

Event	Smokers	Non-Smokers	Statistical Test
*Past tobacco promotional activities*			
I have ever exchanged a cigarette case for a ticket for an entertainment event.	12 (14.1)	14 (4.6)	*χ^2^* = 9.6370, *p* = 0.0019
I have ever exchanged a cigarette case for a prize or on-sale goods.	8 (9.4)	9 (3.0)	*Fisher exact test*, *p* = 0.0164
I have ever participated in an activity sponsored by a cigarette company.	8 (9.4)	11 (3.6)	*Fisher exact test*, *p* = 0.0425
I have ever received free cigarettes during promotional activities.	13 (15.3)	17 (5.6)	*χ^2^* = 8.7852, *p* = 0.0030
*Sources of tobacco advertising in the mass media*			
Billboard			
*Yes*	41 (48.2)	186 (61.2)	*χ^2^* = 4.5832, *p* = 0.0323
Newspaper			
*Yes*	29 (34.1)	140 (46.1)	*χ^2^* = 3.8510, *p* = 0.0497
TV			
*Yes*	35 (41.2)	175 (57.6)	*χ^2^* = 7.1828, *p* = 0.0074
Radio			
*Yes*	20 (23.5)	84 (27.6)	*χ^2^* = 0.5707, *p* = 0.4500
Public transport			
*Yes*	29 (34.1)	130 (42.8)	*χ^2^* = 2.0545, *p* = 0.1518
Poster			
*Yes*	25 (29.4)	100 (32.9)	*χ^2^* = 0.3695, *p* = 0.5433
Goods			
*Yes*	41 (48.2)	174 (57.2)	*χ^2^* = 2.2692, *p* = 0.1320

### 3.4. Smoking-Related Attitude

Table 4 summarizes the comparison of smoking-related attitude between smokers and non-smokers. Smokers were more likely to believe that “Smoking is pleasurable” (*p* < 0.0001), “Smoking is a type of self-presentation” (*p* = 0.0318), “Smoking relaxes me” (*p* < 0.0001), “Smoking makes me look strong” (*p* < 0.0001), “Smoking makes me look mature” (*p* < 0.0001), compared with non-smokers. Moreover, smokers were more inclined to believe that “Smoking makes me confident” (*p* < 0.0001), “Smoking can help me lose weight” (*p* = 0.0295), “Smoking can help me improve my athletic performance” (*p* = 0.0101), and “Smoking can help me study better” (*p* < 0.0001). Smokers were less likely to consider smoking as a waste of money compared with non-smokers (*p* < 0.0001).

**Table 4:** Comparison of smoking-related attitude between smokers and non-smokers (*N*, %).

Attitude	Smokers			Non-Smokers			Statistical Test
	Disagree	Neutral	Agree	Disagree	Neutral	Agree	
Smoking is pleasurable.	27 (31.8)	37 (43.5)	21 (24.7)	246 (80.9)	47 (15.5)	11 (3.6)	*χ^2^* = 83.0153, *p* < 0.0001
Smoking is a type of self-presentation.	65 (76.4)	10 (11.8)	10 (11.8)	263 (86.5)	27 (8.9)	14 (4.6)	*χ^2^* = 6.8938, *p* = 0.0318
Smoking relaxes me.	19 (22.4)	20 (23.5)	46 (54.1)	183 (60.2)	70 (23.0)	51 (16.8)	*χ^2^* = 55.4731, *p* < 0.0001
Smoking makes me look strong.	69 (81.2)	12 (14.1)	4 (4.7)	280 (92.1)	17 (5.6)	7 (2.3)	*χ^2^* = 8.7175, *p* = 0.0128
Smoking makes me look mature.	41 (48.2)	33 (38.9)	11 (12.9)	240 (78.9)	41 (13.5)	23 (7.6)	*χ^2^* = 33.2858, *p* < 0.0001
Smoking makes me confident.	53 (62.4)	23 (27.0)	9 (10.6)	255 (83.9)	41 (13.5)	8 (2.6)	*χ^2^* = 20.9483, *p* < 0.0001
Smoking is a waste of money.	28 (32.9)	26 (30.6)	31 (36.5)	51 (16.8)	42 (13.8)	211 (69.4)	*χ^2^* = 30.8207, *p* < 0.0001
Smoking can help me lose weight.	57 (67.1)	17 (20.0)	11 (12.9)	240 (78.9)	47 (15.5)	17 (5.6)	*χ^2^* = 7.0459, *p* = 0.0295
Smoking can help me improve my athletic performance.	67 (78.8)	14 (16.5)	4 (4.7)	276 (90.8)	21 (6.9)	7 (2.3)	*χ^2^* = 9.1867, *p* = 0.0101
Smoking can help me study better.	53 (62.3)	22 (25.9)	10 (11.8)	266 (87.5)	33 (10.9)	5 (1.6)	*χ^2^* = 33.3740, *p* < 0.0001

### 3.5. Social Pressure on Smoking

Table 5 shows that different sources of social pressure were associated with smoking. Smokers (63.5%) were more likely to believe that friends are the source of pressure to smoking than non-smokers (41.1%) (*p* = 0.002). Smokers (60.0%) were likely to consider schoolmates as source of pressure to smoke than non-smokers (42.1%) (*p* = 0.0090). Moreover, 49.4% of the smokers believed that the pressure to smoke was derived from teachers, while there were no difference regard to teachers as a source of pressure to smoke between smokers and non-smokers(*p* = 0.0861). The difference between smokers and non-smokers in their perspective on smoking as part of celebrations (*p* < 0.0001), public events (*p* < 0.0001), and social activities (*p* < 0.0001) were statistically significant.

### 3.6. Environmental Constraints on Smoking

Table 6 shows the results on environmental constraints on smoking between smokers and non-smokers. The difference between smokers and non-smokers in terms of the difficulty in finding an outdoor smoking place (*p* = 0.0410) was significant. Moreover, the difference in the method used to obtain cigarettes within the past 30 days between smokers and non-smokers was significant (*p* < 0.0001). However, no significant difference was observed between smokers and non-smokers with respect to the difficulty in obtaining cigarettes (*p* = 0.5307) and finding an indoor smoking place (*p* = 0.3194).

**Table 5:** Comparison ofsocial pressure between smokers and non-smokers (*N*, %).

Social Pressure	Smokers	Non-Smokers	Statistical Test
Pressure from friends			
Never	31 (36.5)	179 (58.9)	*χ^2^* = 17.1229, *p* = 0.0018
Few	16 (18.8)	39 (12.8)	
Sometimes	29 (34.1)	52 (17.1)	
Often	5 (5.9)	19 (6.3)	
Always	4 (4.7)	15 (4.9)	
Pressure from schoolmates			
Never	34 (40.0)	176 (57.9)	*χ^2^* = 13.5174, *p* = 0.0090
Few	13 (15.3)	48 (15.8)	
Sometimes	26 (30.6)	46 (15.1)	
Often	5 (5.9)	18 (5.9)	
Always	7 (8.2)	16 (5.3)	
Pressure from teachers			
Never	43 (50.6)	186 (61.2)	*χ^2^* = 8.1541, *p* = 0.0861
Few	18 (21.2)	51 (16.8)	
Sometimes	17 (20.0)	50 (16.4)	
Often	0 (0.0)	7 (2.3)	
Always	7 (8.2)	10 (3.3)	
Smoking brings comfort during celebrations			
Absolutely disagree	11 (12.9)	129 (42.4)	*χ^2^* = 59.1550, *p* < 0.0001
Disagree	18 (21.2)	90 (29.6)	
Neutral	24 (28.2)	57 (18.8)	
Agree	25 (29.4)	26 (8.6)	
Absolutely true	7 (8.2)	2 (0.6)	
Smoking brings comfort in public activities			
Absolutely disagree	15 (17.6)	135 (44.4)	*χ^2^* = 43.0019, *p* < 0.0001
Disagree	23 (27.1)	92 (30.3)	
Neutral	25 (29.4)	57 (18.7)	
Agree	17 (20.0)	19 (6.3)	
Absolutely true	5 (5.9)	1 (0.3)	
Smoking brings comfort in social activities			
Absolutely disagree	13 (15.3)	124 (40.8)	*χ^2^* = 48.0427, *p* < 0.0001
Disagree	13 (15.3)	76 (25.0)	
Neutral	27 (31.8)	73 (24.0)	
Agree	26 (30.6)	27 (8.9)	
Absolutely agree	6 (7.0)	4 (1.3)	

### 3.7. Logistic Regression Model for Identifying Factors that Affect Smoking

We included those variables on socio-demographic data, smoking-related knowledge, attitude, social pressure and environmental constraints with *p* < 0.2 in the bivariate analysis into the logistic regression model. Results of logistic regression analysis with backward elimination indicated that nine factors remained significantly associated with smoking (Table 7). Males were more inclined to smoke than females (OR = 10.837, 95%CI [3.414–34.400]). Respondents who with a living cost of ¥600–800 were less likely to smoke than those with ¥ 600 (OR = 0.185, 95%CI [0.060–0.572]. Respondents were neutral about (OR = 2.0737, 95%CI [1.698–37.257]) the item, “Smoking is pleasurable,” were more likely to smoke than those who disagreed (reference group). Respondents who were neutral about (OR = 7.038, 95%CI [1.987–24.928]) the item “Smoking brings me relax” were more likely to smoke than those who agreed (reference group). Those who agreed with the item “Smoking makes me looked strong” were more likely to smoke than those who disagreed (OR = 19.857, 95%CI [2.066–190.878]). As for the item on “Smoking is a waste of money,” the group that expressed neutrality (OR = 5.488, 95%CI [1.513, 19.905]) or disagreement (OR = 10.171, 95%CI [3.341–30.960]) was more likely to smoke than those who agreed. The neutral attitude toward the item “Smoking could make me a better state in study” was less likely to smoke than those who disagreed (OR = 0.171, 95%CI [0.035–0.840]). The totally disagreed attitude toward the item “Smoking brings comfort on celebration” were less likely to smoke than those who absolutely agreed (OR = 0.033, 95%CI [0.001–0.918]).Considering “Did you get your cigarette in the past 30d?” that those who did not buy any cigarettes were less likely to smoke than respondents who brought cigarettes (OR = 0.059, 95%CI [0.024–0.145]).

**Table 6:** Comparison of the environmental constraints between smokers and non-smokers (*N*, %).

Variables	Smokers	Non-Smokers	Statistical Test
Difficulty in obtaining cigarettes			
Very difficult	2 (2.4)	16 (5.3)	*Fisher exact test*, *p* = 0.5307
Difficult	3 (3.5)	19 (6.3)	
Easy	33 (38.8)	102 (33.5)	
Very easy	47 (55.3)	167 (54.9)	
How did you obtain cigarette in the past 30 days?			
Do not buy any cigarette	19 (22.4)	273 (89.8)	*Fisher exact test*, *p* < 0.00001
Buy from store	55 (64.7)	17 (5.6)	
With the help of somebody	3 (3.5)	0 (0.0)	
Other ways	8 (9.4)	14 (4.6)	
Difficulty in finding an indoor smoking place?			
Very difficult	11 (12.9)	40 (13.2)	*χ^2^* = 3.5107, *p* = 0.3194
Difficult	18 (21.2)	94 (30.9)	
Easy	35 (41.2)	101 (33.2)	
Very easy	21 (24.7)	69 (22.7)	
Difficulty in finding an outdoor smoking place?			
Very difficult	5 (5.9)	16 (5.3)	*χ^2^* = 8.2555, *p* = 0.0410
Difficult	12 (14.1)	26 (8.5)	
Easy	38 (44.7)	103 (33.9)	
Very easy	30 (35.3)	159 (52.3)	

## 4. Discussion

Despite good knowledge on the hazards of tobacco, 21.85% of fresh undergraduates in this study reported they smoked which was similar to a previous study among college students [5] but higher than the rate among individuals aged 15–25 at the national level [4]. Adolescence is believed to be a high-risk period for starting to smoke, and fresh undergraduates are in that period. The current study indicates that fresh undergraduates were more likely to smoke to achieve relaxation and self-presentation. This might be due to that numerous college students, particularly male students, experience changes in self-consciousness and interpersonal relationships, and hence the desire to be recognized, just as they believe smoking is associated with independence and maturity [22]. Administrators should focus more on smoking psychology and behavior of college students to help manage the latter’s smoking behaviors [23]. Efforts should be taken to educate undergraduates on effective strategies in managing stress during their course, as they thought that tobacco smoking could be used as a coping strategy to face such stress [24]. For instance, teachers can motivate students to adopt a positive attitude toward life, and offer classes that teach ways of eliminating negativity when under stress [25].

**Table 7:** Logistic regression model for identifying factors that affect smoking (*N* = 389).

Variable	OR	(95%CI)	*p*-Value
*Demographic data*			
Sex			
Female	1		<0.0001
Male	10.837	(3.414, 34.400)	
Living cost			
<¥600	1	--	--
¥600–¥800	0.185	(0.060, 0.572)	0.0034
>¥800	0.828	(0.293, 2.335)	0.7209
*Smoking-related attitude*			
Smoking is pleasurable			
Disagree	1	--	--
Neutral	7.954	(1.698, 37.257)	0.0085
Agree	2.819	(0.609, 13.044)	0.1848
Smoking relaxes me			
Disagree	1	--	--
Neutral	7.038	(1.987, 24.928)	0.0025
Agree	1.080	(0.358, 3.256)	0.8911
Smoking makes me look strong			
Disagree	1	--	--
Neutral	2.198	(0.487, 9.916)	0.3057
Agree	19.857	(2.066, 190.878)	0.0096
Smoking is a waste of money			
Agree	1	--	--
Neutral	5.488	(1.513, 19.905)	0.0096
Disagree	10.171	(3.341, 30.960)	<0.0001
Smoking can help me study better			
Disagree	1	--	--
Neutral	0.171	(0.035, 0.840)	0.0296
Agree	3.305	(0.978, 11.164)	0.0542
*Social pressure*			
Smoking brings comfort during celebration			
Absolutely agree	1	--	--
Agree	1.645	(0.460, 5.882)	0.4437
Neutral	0.356	(0.104, 1.221)	0.1006
Disagree	0.343	(0.094, 1.245)	0.1037
Absolutely disagree	0.033	(0.001, 0.918)	0.0444
*Environmental constraints*			
How did you get your cigarettes in the past 30 days?			
Brought cigarettes	1	--	--
Did not buy any cigarette	0.059	(0.024, 0.145)	<0.0001

Note: Abbreviation: CI confidence intervals, OR odds ratio.

In terms of the influence encountered in the school environment, in line with previous studies [26], we found that social pressure was an important associated factor of smoking among our respondents. Mutual cigarette smoking phenomenon is likewise common, particularly in the bedroom as a unit of aggregation [27]. Our study found that smokers received more pressure from classmates, friends compared with non-smokers, which are to be in favor of another survey in China [28]. Moreover, smoking is a social activity, and studies have highlighted the social aspect of smoking and its strong association with social interaction [29]. Tobacco control activities may be popularized to equip people with the skills to refuse smoking and effectively resist all types of cigarette-related behaviors [30]. Positive mutual communication among students and raising their awareness to consciously resist tobacco may be promoted as well. Students prefer to smoke in bars and on campus as they regard smoking in public as impolite [22]. In the present study, smokers and non-smokers have been found to have easy access to cigarettes and can readily find an indoor smoking place. The easy availability of tobacco could promote smoking among college students. Some smokers may find the increased difficulty in purchasing tobacco as a stimulus to break the habit [31]. In addition, we found that neutrality and disagreement to “Smoking is a waste of money” is a risk factor of smoking in the current study suggests that spending money for cigarettes may not be a barrier for smoking in China, and prohibited shops selling cigarettes in the school may be a way of decreasing the source of tobacco. Although non-significant in the logistic regression, bivariate tests showed that significantly more non-smokers reported that they were exposed to three out of the seven sources of tobacco advertisements (via billboard, newspaper and TV) than smokers. The higher proportions of non-smokers with higher level of tobacco advertisement exposure might be because non-smokers were more sensitive to tobacco atmosphere [32]. The implication of such findings is that WHO Framework Convention on Tobacco Control (FCTC) policies should pay attention to the restriction of retail sales outlets near universities in the future. In the future, we really hope that the Chinese government and other countries government agencies will implement the relevant policies. Smokers experience difficulty in finding an outdoor smoking area. May be a voluntary health origination should make efforts in against tobacco use and the restriction of smoking in public places, and it is important that smoke-free policy and buildings exist on campus as well [33].

Logistic regression analysis also indicated that male college students were more likely to smoke than female. This study further demonstrates that among college students in China, male smokers outnumber female smokers [34]. In future, tobacco control education continuously pays more attention male college students. Similar to many previous studies in tobacco control [6,8,9,13,14,32,35], we also found that students adopted the attitude that smoking is pleasurable, and they believe that smoking is not a waste of money, smoking brings me relax, smoking makes me looked strong, smoking could make me a better state in study, and smoking brings comfort on celebration, were more inclined to smoke in our study. The findings indicate changing college students’ smoking related concepts and cognition are very important to tobacco control. Our study found that those undergraduates who do not buy any cigarettes are less likely to smoke than respondents who buy cigarettes. This result further supports the importance of the restriction of retail sales outlets and tobacco promotion activities near universities.

This study also has certain limitations. Firstly, this study only included students majoring in education and medicine from two universities in the Chongqing University Town, and hence the current findings may not be generalizable to the whole population of fresh undergraduates in China. Further studies with a larger sample size which covers students in other disciplines are needed. Secondly, in the current study, majority of the participants were non-smokers and most of the smokers were male, and hence it is possible that the observed differences in knowledge, attitude, social pressure and environmental constraints between smokers and non-smokers are because of the high smoking prevalence in male students [36]. Thirdly, the information regarding smoking status was collected by self-reports which might have introduced response bias to the current findings. Since it is commonly agreed that smoking is an unhealthy habit and it is expected that students who smoke might have reported they were non-smokers due to social desirability. Consequently, the smoking prevalence among fresh undergraduates might have been underestimated in the current sample. Finally, owing to the cross-sectional survey data used in the current study, no causal inferences regarding the results could be made.

## 5. Conclusions

Our study found that 21.85% of fresh undergraduates were smokers. Our study shows that smoking-related behavior was associated with smoking-related attitude, social pressure, and environmental constraints. This study provides more detailed consideration of the implications for the WHO Framework Convention on Tobacco Control (FCTC) policies, especially on restriction of retail sales outlets and tobacco promotion activities near universities would be valuable. Our findings also provide a knowledge base from which to develop targeted tobacco control policies for fresh undergraduates. Such smoking prevention program may focus on modifying attitudes towards smoking and providing a cigarette-free environment near the campus.
